# Ethnic Differences in Mammographic Densities: An Asian Cross-Sectional Study

**DOI:** 10.1371/journal.pone.0117568

**Published:** 2015-02-06

**Authors:** Shivaani Mariapun, Jingmei Li, Cheng Har Yip, Nur Aishah Mohd Taib, Soo-Hwang Teo

**Affiliations:** 1 Cancer Research Initiatives Foundation, Sime Darby Medical Centre, 1 Jalan SS12/1A, Subang Jaya, 47500 Selangor, Malaysia; 2 Breast Cancer Research Unit, University Malaya Cancer Research Institute, Faculty of Medicine, University of Malaya, 50603, Kuala Lumpur, Malaysia; 3 Human Genetics, Genome Institute of Singapore, Singapore 138672, Singapore; 4 Sime Darby Medical Centre, 1 Jalan SS12/1A, Subang Jaya, 47500 Selangor, Malaysia; Central South University, CHINA

## Abstract

**Background:**

Mammographic density is a strong risk factor for breast cancer and is highly variable, but, to date, few studies have examined density in Asian women, particularly those in low and middle-income Asian countries where genetic and lifestyle determinants may be significantly different.

**Methods:**

A total of 1,240 women who attended an opportunistic mammogram screening programme were eligible for analysis. Mammographic density was estimated using a fully-automated thresholding method and differences across ethnic groups were examined using linear regression in 205 randomly selected Chinese women, 138 Malay and 199 Indian women.

**Results:**

Percent density was significantly higher in Chinese women (28.5%; 95% CI 27.0%, 30.0%) compared to Malay (24.2%; 95% CI 22.5%, 26.0%) and Indian (24.3%; 95% CI 22.8%, 25.7%) women (p<0.001), after adjustment for age, BMI, menopausal status, parity and age at first full term pregnancy. Correspondingly, adjusted nondense area was significantly lower in Chinese (72.2cm^2^; 95% CI 67.9cm^2^, 76.5cm^2^) women compared to Malay (92.1cm^2^; 95% CI 86.9cm^2^, 97.2cm^2^) and Indian (97.7cm^2^; 95% CI 93.4cm^2^, 101.9cm^2^) women (p<0.001), but dense area did not differ across the three ethnic groups.

**Conclusions:**

Our study shows that higher percent density and lower nondense area reflect the higher incidence of breast cancer in Chinese compared to Malay and Indian women in Malaysia. Known lifestyle determinants of mammographic density do not fully account for the ethnic variations observed in mammographic density in this Asian cohort.

## Introduction

Mammographic density is the area on a mammogram that is white and represents the radiodense connective and epithelial tissue in the breast. Mammographic density is expressed either as dense area, which in cm^2^, is the total area that is radiodense, or percent mammographic density, which is the proportion of dense tissue to the total amount of breast tissue. Notably, percent mammographic density and absolute dense area have been shown to be strong risk factors for breast cancer, where women with highest densities have an increased risk of up to six-fold compared to those with little or no densities [1,2,3,4,5]. Nondense areas of the mammogram, which represent adipose tissue, on the other hand have been suggested to be protective against breast carcinogenesis [6].

Although ethnic differences in mammographic density have been studied, the majority of these have been amongst multi-ethnic cohorts in Western countries, such as the United States and the United Kingdom. These studies show that percent density and absolute dense area reflect the differences in breast cancer incidence rates in Asian compared to Caucasian women [7,8,9]. Few studies have examined mammographic density in Asians, particularly those living in low and middle income Asian countries. The majority of Asian countries practice opportunistic screening [10] and organised population-based screening are only available in high income Asian countries such as Singapore, Japan and Korea [11].

In Malaysia, the incidence of breast cancer differs across the three major ethnic groups. Chinese women have the highest age standardised incidence rate, with 59.7 per 100 000 followed by the Indians and Malays at 55.8 and 33.9 respectively [12,13]. Mammographic density has been described previously in Chinese populations in Asia [14,15] but not yet in Malays and Indians living in Asia. Malay and Indian women constitute a significant proportion of women in the Malay Archipelago and the Indian subcontinent and make up 51% and 8% of the Malaysian population, respectively. This gives us the opportunity to study ethnic differences and lifestyle determinants of mammographic density in these ethnic groups, especially in the current generation before the disease risk approaches that of Caucasian populations through Westernisation and urbanisation. We sought to investigate whether the differences in percent density, dense area and nondense area across these three ethnicities reflect the variation in breast cancer incidence rates.

## Methods

### Study population and data collection

A cross-sectional study was conducted among participants of the Malaysian Mammogram Study (MyMammo), one of the several subsidised opportunistic mammogram screening programmes in the country. Women between 40 and 74 years of age with no personal history of breast cancer were eligible to participate. Participants were recruited using flyers, posters and articles in the mainstream media. Although information on the subsidised programme was provided equally across the English, Chinese and Malay media, the majority of participants were Chinese. All women provided written informed consent and this study was approved by the Sime Darby Medical Centre Independent Ethics Committee.

The questionnaire included information on anthropometric factors, menstrual and reproductive history, family history of cancer and dietary practices. Anthropometric information was self-declared for about 30% of the subjects and measured by the interviewer for the remaining 70%. Analyses conducted by stratifying the subjects by self-reported and measured height and weight showed no impact on the results of this study.

Mammograms were available for 1603 of the 1646 women who participated in MyMammo between October 2011 and October 2013. Women were excluded from this analysis if they were of mixed ethnicities or ethnicities other than Malay, Indian and Chinese (n = 84). Where women were known to be related, the oldest participant was included in the analysis. Of the 1356 unrelated participants, we excluded women who have had silicone injections to their breasts (n = 3), were symptomatic (n = 91) or were diagnosed with cancer (n = 8), had missing BMI data (n = 16) or never had menstrual periods (n = 1). Of the 1240 eligible women for analysis, there were 138 Malays, 199 Indian and 903 Chinese.

### Mammographic density assessment

Mammograms were performed with the Hologic Selenia full field digital mammography (FFDM) system. Mammographic density measurements of the processed images were performed using a fully-automated thresholding method based on the ImageJ software, as described in [16]. Measurements in pixels were converted into cm^2^ using the conversion factor obtained from the images. Nondense areas were calculated by subtracting dense area from total breast area.

Correlation coefficients revealed a strong positive correlation between percent mammographic density estimated by the ImageJ method and Cumulus measurements performed by a trained reader (JL) for 50 randomly selected validation images. Concordance correlation and Bland-Altman plots are shown in S1 and S2 Figs. A validation study was also performed for randomly selected images within each ethnicity. Bland-Altman plots for the ethnicity stratified validation study are shown in S3, S4 and S5 Figs. Concordance correlation and Bland-Altman plots were created using R.

The CC and MLO view measurements were strongly correlated but the means were significantly different. There was no difference between measurements from the left and right mammograms. Statistical analyses were performed using randomly selected left or right CC view mammograms.

### Statistical Analyses

BMI was calculated by dividing weight (kg) by the square of the height (m). Subjects were categorised as parous if they have had at least one full term pregnancy (live or still births). Menopausal status was divided into two groups i.e. premenopausal or perimenopausal and postmenopausal. Postmenopausal status was defined as no menses for the past one year. For subjects who did not provide date of last menses, the difference between the age at menopause and age at consent was calculated and those with a difference of 1 or more were categorised as postmenopausal. There were 19 women who have had bilateral oophorectomies, of whom 17 had the surgery before and 2 after menopause. Ever use of oral contraceptives and hormone replacement therapy was defined as at least one month of usage. The analyses for green tea and black tea consumption excluded those who consumed different types of tea regularly. Soy intake included consumption of soy milk, tofu, fermented soy beans and other soy products. Family history of breast cancer in a first degree relative included affected mothers and sisters.

Differences in the subject characteristics across ethnicities were determined using chi-square and F tests. We identified the determinants of percent density, dense area and nondense area using linear regression. In the regression models, we assessed the relationships of a priori determinants of mammographic density and the known risk factors for breast cancer. The regression coefficients for weight and height in the multivariable adjusted models were calculated after excluding BMI from the models. We used methods described previously [15] to recode variables which are applicable only to parous subjects in the multivariable adjusted model. Briefly, the regression coefficient for age at first full term pregnancy represents the effect of a one year difference in age at first full term pregnancy among parous subjects. The regression coefficient for number of full term pregnancies estimates the effect of additional full term pregnancies for parous subjects who have had more than one full term pregnancy. Variables significantly associated with density (*p*<0.05) in the multivariable ethnicity adjusted analysis were adjusted for in subsequent analyses. We compared the unadjusted and adjusted estimated marginal means of percent density, dense area and nondense area across ethnicities using linear regression and analysis of covariance models, respectively. Covariates were selected based on the determinants of density that were identified in previous regression analyses. Pairwise comparisons of ethnic groups were made using the Sidak test. We repeated the analysis after stratifying the cohort by menopausal status. We also repeated the age and BMI adjusted analyses upon stratifying the subjects into two groups; self-reported and measured anthropometric measurements.

All regression models were checked for normality of residuals and no data transformations were necessary. Linearity, homoscedasticity and independence of residuals were inspected for all continuous independent variables. Models were inspected for multicollinearity among the independent variables. The homogeneity of variance assumption for the analysis of covariance was affected by unequal sample sizes across the three ethnic groups [138 Malays, 199 Indian and 903 Chinese]. We therefore repeated all analyses using a randomly selected sample of 205 Chinese women for meaningful comparisons across ethnicities [<50% greater than the smallest cohort i.e. 138 Malays]. Selected characteristics of the random sample compared to all Chinese women are presented in S1 Table. All statistical analyses were performed using Statistical Package and Service Solutions (SPSS) Version 16.0.

## Results

### Study cohort characteristics

The characteristics of all subjects used in this study stratified by ethnicity are presented in Table 1. The mean age was youngest for Malays (48.4 years) compared to Chinese (50.8 years) and Indians (51.4 years). Malays and Indians had 14% higher BMI than the Chinese. The majority of women (86.9%) were parous and Malay women had the most pregnancies and breastfed the most frequently and for the longest duration. Malay women were more likely to have used oral contraceptives [40.2% compared to 25.6% Chinese and 23.6% Indian], whereas Indian women were more likely to have used hormone replacement therapy. Consistent with the population risk to breast cancer, more Chinese women, followed by Indian women, had a family history of breast cancer compared to Malay women [13%, 12.2% and 4.4% respectively]. Few women were current smokers and there was a higher proportion of ever and current smokers among Malay women. More Chinese and Indian women consumed alcohol. Women in our cohort were more affluent than the national average (48% versus 66% earning less than RM5000 per month) and more likely to have at least secondary education (93% versus 62%) compared to the general Malaysian population [17,18]. In particular, the Malay women who participated in the opportunistic screening programme were more highly educated and affluent than the Indian or Chinese women.

**Table 1:** Characteristics of subjects.

Characteristic	All (*N* = 542)		Malay (*n* = 138)		Indian (*n* = 199)		Chinese (*n* = 205)		*p* value^a^
	Mean (SD)	%	Mean (SD)	%	Mean (SD)	%	Mean (SD)	%	
Age (years)	50.4 (7.4)		48.4 (6.3)		51.4 (7.2)		50.8 (7.9)		<0.001
*Anthropometric*									
Height (cm)	157.1 (5.9)		156.0 (5.5)		157.7 (6.2)		157.2 (5.9)		0.032
Weight (kg)	63.6 (12.4)		65.9 (13.0)		67.3 (12.5)		58.6 (10.2)		<0.001
BMI (kg/m^2^)	25.8 (4.9)		27.0 (4.5)		27.2 (5.3)		23.7 (4.0)		<0.001
*Menstrual and reproductive*									
Age at menarche (years), *n* = 539	12.9 (1.4)		12.9 (1.4)		12.9 (1.4)		12.8 (1.3)		0.896
Parity (Parous)		86.9		87.7		89.9		83.4	0.143
Number of FTP	2. 4 (1.4)		2.8 (1.7)		2.4 (1.3)		2.1 (1.3)		<0.001
Age at FFTP^b^ (years)	27.4 (4.8)		26.6 (4.4)		27.0 (4.9)		28.2 (5.0)		0.012
Breastfeeding^b^, *n* = 465									
Ever		82.8		94.9		87.1		69.8	<0.001
Duration (months)									
0		17.2		5.1		13.0		30.2	
>0–12		53.7		34.7		60.5		59.7	
>12		29.1		60.2		26.6		10.1	
Multiple 12 month breastfeeding^b^		12.3		32.2		9.0		1.8	<0.001
Menopausal status (post)		42.1		30.4		50.8		41.5	0.001
Age at menopause^c^ (years), *n* = 219	49.1 (4.8)		49.5 (4.0)		48.3 (5.4)		50.0 (4.1)		0.043
Bilateral oophorectomy (yes), *n* = 541		3.5		2.2		3.5		4.4	
*Exogenous hormones*									
Oral contraceptives use									
Ever, *n* = 534		28.5		40.2		23.6		25.6	0.003
Current, *n* = 526		1.3		3.0		0		1.5	0.067
Duration of use (months), *n* = 534									
0		71.5		59.8		76.4		74.4	
1–11		7.5		5.3		6.5		9.9	
≥12		21.0		34.9		17.1		15.7	
Hormone therapy use									
Ever, *n* = 537		7.6		4.4		11.2		6.4	0.050
Current, *n* = 540		3.5		1.4		6.6		2.0	0.013
Duration of use (months), *n* = 537									0.105
0		92.4		95.6		88.8		93.6	
1-<6		2.6		0.7		3.6		3.0	
6-<24		0.9		0.7		1.0		1.0	
≥24		4.1		2.9		6.6		2.5	
*Lifestyle variables*									
Smoking									
Ever		7.7		15.9		2.0		7.8	<0.001
Current		2.0		5.1		0.5		1.5	0.011
Alcohol (>once a month), *n* = 540		18.7		5.1		22.1		24.6	<0.001
Coffee (≥one cup a day), *n* = 541		61.0		55.1		67.3		58.8	0.055
Green tea (≥one cup a day), *n* = 422		7.1		7.8		7.7		6.1	0.812
Black tea (≥one cup a day), *n* = 422		28.4		37.3		38.1		13.9	<0.001
Soy products (daily), *n* = 538		10.0		10.2		11.1		8.9	0.754
*Other variables*									
FHBC 1^st^ degree relative, *n* = 525		10.5		4.4		12.2		13.0	0.029
Educational level, *n* = 535									<0.001
Primary or less		6.9		0.7		8.6		9.5	
Secondary		48.8		32.1		57.4		51.7	
Tertiary		44.3		67.2		34.0		38.8	
Average monthly household income, *n* = 536									<0.001
<RM5000		47.9		32.6		57.1		49.5	
RM5000-10 000		29.5		30.4		30.3		28.0	
>RM10 000		22.5		36.9		12.6		22.5	

Abbreviations: BMI body mass index, FFTP first full term pregnancy, FTP full term pregnancy, FHBC family history of breast cancer

^a^ *p* value from F test for continuous variables and χ^2^ for categorical variables, comparing ethnic groups.

^b^ Restricted to parous women

^c^ Restricted to postmenopausal women

### Determinants of percent density, dense area and nondense area

Older age at mammogram was significantly associated with a decrease in percent density in all ethnicities. In the age and mutually adjusted regression analysis, we found that percent density decreased with BMI, parity status, earlier age at first full term pregnancy, multiple 12 month breastfeeding and postmenopausal status (S2 Table). After adjusting for ethnicity, all variables mentioned above remained significantly associated with percent density in the same direction, with the exception of multiple 12 month breastfeeding. Taken together, age, BMI, menopausal status, parity, age at first full term pregnancy and ethnicity explained 45.4% of the variation in percent density.

Dense area was found to reduce with increasing BMI, parity status and postmenopausal status in the age and mutually adjusted analysis before and after adjusting for ethnicity (S3 Table). Current use of HRT in postmenopausal women was associated with smaller dense area in the ethnicity adjusted analysis, although it is to be noted that there are only few current HRT users in the study. Taken together, age, BMI, menopausal status and parity accounted for 16.1% of the variation in dense area.

Nondense area was positively associated with BMI and postmenopausal status in the fully adjusted regression analysis (S4 Table). Taken together, age, BMI and menopausal status explained 45.8% of variation in nondense area.

Variables associated with socioeconomic status i.e. education level and average household income were not associated with percent density and dense area in all the adjusted models. We did not find an association between education level and nondense area after adjusting for age, BMI and menopausal status but did find a significant negative association after including ethnicity into this model, suggesting that this association is mediated by the association between ethnicity and nondense area. Income level was not associated with nondense area in all adjusted analyses.

None of the dietary variables i.e. soy, alcohol, coffee and tea intake, were associated with mammographic density. Similarly, we did not observe an association between density and ages at menarche and menopause, having a bilateral oophorectomy, breastfeeding (among parous women), use and duration of use of oral contraceptives and hormone replacement therapy, smoking and having an affected first degree relative (S2, S3 and S4 Tables).

### Ethnic differences in mammographic density

**Fig 1:**
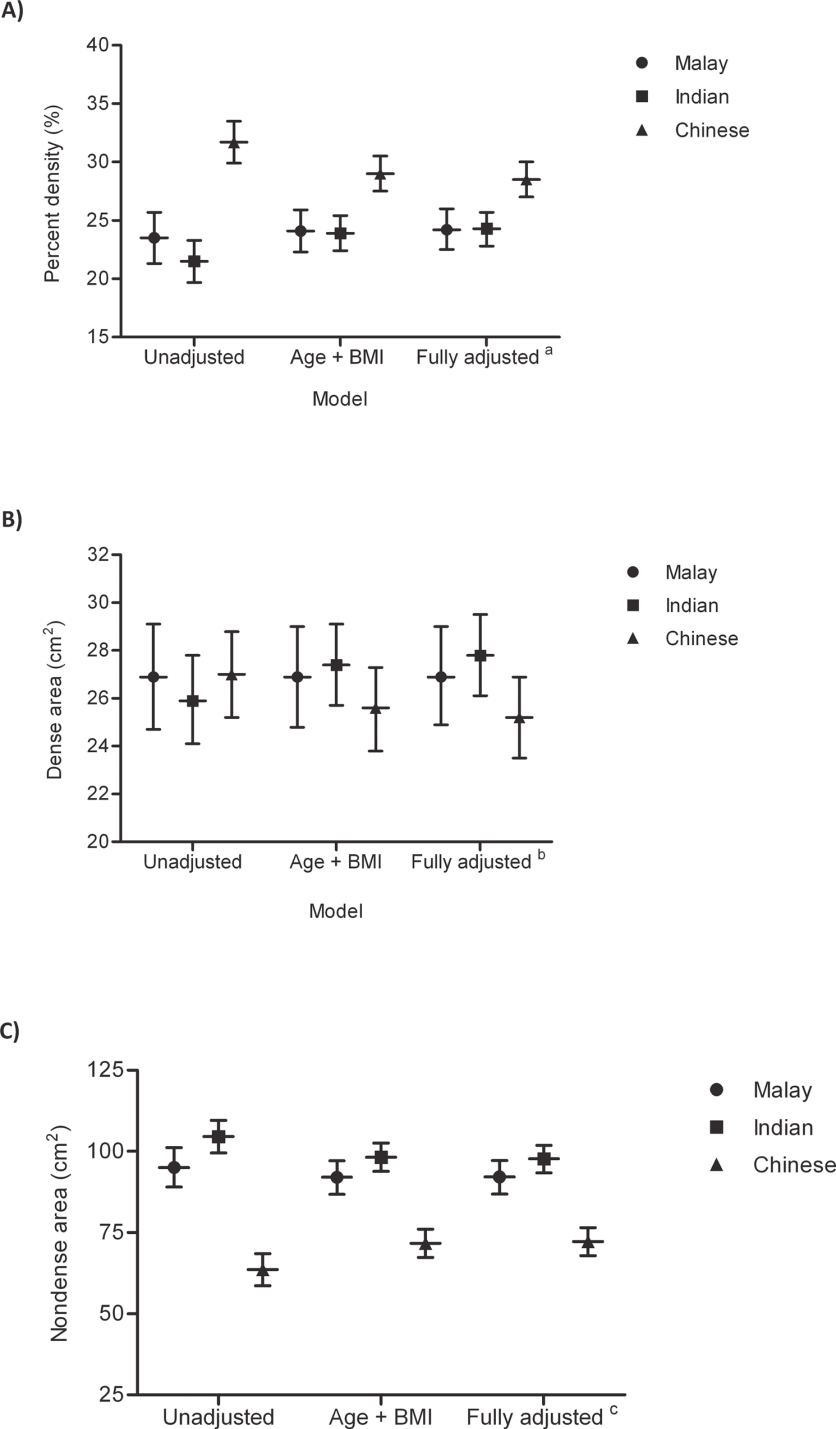
Unadjusted and adjusted A) mean percent density, B) mean dense area and C) mean nondense area with 95% confidence intervals by ethnicity. Note: ^a^Adjusted for age, BMI, menopausal status, parity and mean adjusted age at first full term pregnancy; ^b^Adjusted for age, BMI, menopausal status and parity; ^c^Adjusted for age, BMI and menopausal status.

Fig. 1 shows that percent density is higher and non-dense area is lower in Chinese compared to Malay and Indian women [distribution of percent density by ethnicity is shown in S6 Fig.]. In order to determine whether the ethnic differences in mammographic density were mediated by differences in lifestyle variables, we conducted an analysis by adjusting for factors associated with each measure of mammographic density [Table 2]. Notably, the variables associated with each measure of mammographic density is different, with only BMI and post-menopausal status associated with all 3 measures of mammographic density. Chinese women had 35% and 47% higher percent density compared to Malay and Indian women respectively. This difference was partly accounted for by the lower BMI in Chinese women and reduced to 20% and 21% higher percent density respectively after adjusting for age and BMI. Further adjustment for menopausal status, parity and age at first full term pregnancy marginally reduced this difference to 18% and 17%. Percent density did not differ between Malay and Indian women in the unadjusted (*p* = 0.42) and fully adjusted (*p* = 1.00) models. Chinese women had 33% and 39% smaller nondense area compared to Malay and Indian women respectively. This difference was partly explained by the lower BMI observed in Chinese women compared to women of Malay and Indian ethnicities. After adjusting for age and BMI Chinese women had 22% and 27% less nondense area compared to Malay and Indian women respectively and further adjustment for menopausal status only reduced these differences by less than 1%. Nondense areas were similar in Malay and Indian women. There were no significant differences in dense areas across the three ethnic groups in both unadjusted and adjusted analyses.

**Table 2:** Unadjusted and adjusted mean percent mammographic density, dense area and nondense area in Malay, Indian and Chinese women, Malaysia, 2011–2013.

Model	Malay (*n* = 138)		Indian (*n* = 199)		Chinese (*n* = 205)		Overall *p* value	Multiple comparisons *p* value^a^		
	Mean	95% CI	Mean	95% CI	Mean	95% CI		Malay vs. Indian	Malay vs. Chinese	Indian vs. Chinese
*Percent density (%)*										
Unadjusted	23.5	21.3, 25.7	21.5	19.7, 23.3	31.7	29.9, 33.5	<0.001	0.415	<0.001	<0.001
Adjusted for age + BMI	24.1	22.3, 25.9	23.9	22.4, 25.4	29.0	27.5, 30.5	<0.001	0.995	<0.001	<0.001
Fully adjusted^b^	24.2	22.5, 26.0	24.3	22.8, 25.7	28.5	27.0, 30.0	<0.001	1.000	<0.001	<0.001
*Dense area (cm^2^)*										
Unadjusted	26.9	24.7, 29.1	25.9	24.1, 27.8	27.0	25.2, 28.8	0.680	0.893	0.999	0.791
Adjusted for age + BMI	26.9	24.8, 29.0	27.4	25.7, 29.1	25.6	23.8, 27.3	0.350	0.981	0.714	0.401
Fully adjusted^c^	26.9	24.9, 29.0	27.8	26.1, 29.5	25.2	23.5, 26.9	0.131	0.898	0.534	0.132
*Nondense area (cm^2^)*										
Unadjusted	95.0	89.0, 101.1	104.5	99.5, 109.5	63.6	58.6, 68.5	<0.001	0.055	<0.001	<0.001
Adjusted for age + BMI	92.0	86.8, 97.1	98.2	93.9, 102.5	71.7	67.4, 76.0	<0.001	0.186	<0.001	<0.001
Fully adjusted^d^	92.1	86.9, 97.2	97.7	93.4, 101.9	72.2	67.9, 76.5	<0.001	<0.001	<0.001	<0.001

Abbreviations: BMI body mass index, CI confidence interval, FFTP first full term pregnancy

^a^ *p* value from Sidak test.

^b^ Adjusted for variables associated with percent density [age, BMI, parity, age at first full term pregnancy and menopausal status]

^c^ Adjusted for variables associated with dense area [age, BMI, parity, menopausal status]

^d^ Adjusted for variables associated with non-dense area [age, BMI, menopausal status]

We found results that were similar to those described above when we analysed only premenopausal women (Table 3). Chinese women had significantly higher percent density and smaller nondense area compared to Malay and Indian women; percent density and nondense area did not differ between Malay and Indian women; and there were no differences in absolute density across all three ethnicities. In postmenopausal women (Table 4), Chinese women had higher percent density compared to women of other ethnicities but this difference was no longer significant after further adjusting for variables associated with percent density [parity and age at first full term pregnancy]. As observed among premenopausal women, dense area did not differ across the ethnic groups and nondense areas were significantly smaller in the Chinese compared to Malays and Indians among postmenopausal women.

**Table 3:** Unadjusted and adjusted mean percent mammographic density, dense area and nondense area in premenopausal Malay, Indian and Chinese women, Malaysia, 2011–2013.

Model	Malay (*n* = 90)		Indian (*n* = 98)		Chinese (*n* = 120)		Overall *p* value	Multiple comparisons *p* value^a^		
	Mean	95% CI	Mean	95% CI	Mean	95% CI		Malay vs. Indian	Malay vs. Chinese	Indian vs. Chinese
*Percent density (%)*										
Unadjusted	25.2	22.7, 27.7	25.2	22.7, 27.7	36.0	33.7, 38.2	<0.001	1.000	<0.001	<0.001
Adjusted for age + BMI	27.3	25.1, 29.5	27.3	25.2, 29.4	32.6	30.6, 34.6	<0.001	1.000	0.002	0.002
Fully adjusted^b^	27.6	25.5, 29.8	27.3	25.2, 29.4	32.3	30.3, 34.3	0.002	0.994	0.010	0.003
*Dense area (cm^2^)*										
Unadjusted	28.7	26.1, 31.3	29.2	26.6, 31.8	30.2	27.8, 32.6	0.690	0.988	0.784	0.931
Adjusted for age + BMI	29.9	27.4, 32.5	30.5	28.0, 33.0	28.2	25.8, 30.5	0.392	0.982	0.701	0.461
Fully adjusted^c^	30.1	27.6, 32.7	30.5	28.0, 33.0	28.0	25.7, 30.3	0.323	0.995	0.564	0.409
*Nondense area (cm^2^)*										
Unadjusted	92.5	85.5, 99.6	95.8	88.8, 102.8	56.6	50.3, 62.9	<0.001	0.887	<0.001	<0.001
Adjusted for age + BMI^d^	86.5	80.3, 92.8	90.5	84.4, 96.7	65.8	60.0, 71.5	<0.002	0.747	<0.001	<0.001

Abbreviations: BMI body mass index, CI confidence interval, FFTP first full term pregnancy

^a^ *p* value from Sidak test.

^b^ Adjusted for variables associated with percent density [age, BMI, parity and age at first full term pregnancy]

^c^ Adjusted for variables associated with dense area [age, BMI and parity]

^d^ Adjusted for variables associated with non-dense area [age and BMI]

**Table 4:** Unadjusted and adjusted mean percent mammographic density, dense area and nondense area in postmenopausal Malay, Indian and Chinese women, Malaysia, 2011–2013.

Model	Malay (*n* = 42)		Indian (*n* = 101)		Chinese (*n* = 85)		Overall *p* value	Multiple comparisons *p* value^a^		
	Mean	95% CI	Mean	95% CI	Mean	95% CI		Malay vs. Indian	Malay vs. Chinese	Indian vs. Chinese
*Percent density (%)*										
Unadjusted	19.7	16.0, 23.3	17.9	15.6, 20.3	25.6	23.1, 28.2	<0.001	0.808	0.025	<0.001
Age + BMI	19.8	16.8, 22.9	19.7	17.7, 21.7	23.5	21.2, 25.7	0.038	1.000	0.172	0.048
Fully adjusted^b^	19.5	16.5, 22.5	20.0	18.0, 21.9	23.3	21.1, 25.5	0.053	0.992	0.138	0.092
*Dense area (cm^2^)*										
Unadjusted	22.7	19.0, 26.4	22.7	20.4, 25.1	22.5	19.9, 25.1	0.993	1.000	1.000	0.999
Adjusted for age + BMI	22.7	19.1, 26.3	23.6	21.3, 26.0	21.5	18.9, 24.1	0.493	0.965	0.927	0.552
Fully adjusted^c^	22.0	18.5, 25.6	24.2	21.9, 26.6	20.9	18.4, 23.5	0.183	0.668	0.947	0.198
*Non-dense area (cm^2^)*										
Unadjusted	100.7	89.8, 111.7	112.9	105.9, 120.0	73.4	65.7, 81.1	<0.001	0.186	<0.001	<0.001
Adjusted for age + BMI^d^	99.9	90.9, 108.9	107.1	101.2, 113.0	80.7	74.2, 87.2	<0.001	0.464	0.003	<0.001

Abbreviations: BMI body mass index, CI confidence interval, FFTP first full term pregnancy

^a^ *p* value from Sidak test.

^b^ Adjusted for variables associated with percent density [age, BMI, parity and age at first full term pregnancy]

^c^ Adjusted for variables associated with dense area [age, BMI, parity and current use of hormone replacement therapy]

^d^ Adjusted for variables associated with non-dense area [age and BMI]

## Discussion

Our study shows that percent mammographic density and nondense area, but not dense area, vary amongst Asians of different ethnicities living in the same region. Chinese women have significantly higher percent mammographic density and lower nondense area compared to Malays and Indians. Notably, in post-menopausal women, age, BMI and parity explained the differences in percent density across ethnic groups. By contrast, in pre-menopausal women, age, BMI and parity did not account for ethnic differences, suggesting that the mechanism by which percent density is modulated in post-menopausal women may be different from that in premenopausal women. These results suggest that genetic or other hitherto undetermined factors may explain the remaining variation observed across these ethnic groups. To the best of our knowledge, our study is the first to investigate ethnic variations in mammographic density measured as a continuous variable and with adjustment for lifestyle variables among Asian women living in Asia.

Other studies have shown that ethnic differences in percent mammographic density and dense area reflect variations in breast cancer risk [9,19,20]. Consistent with this, we found that both breast cancer risk and percent density are highest amongst Chinese women compared to the Indians and Malays, reflecting the higher breast cancer risk in Chinese. However, although Indian women in Malaysia have higher age standardised incidence rates of breast cancer compared to Malay women, we did not find any difference between percent densities of Indians compared to Malays in our study. One possible explanation is that the Malay women in our study, who were more affluent and well-educated compared to the other two ethnicities and to the Malaysian population, probably had lower BMI and higher percent density compared to the rest of the Malays in Malaysia. Indeed, other studies have reported that poorer socioeconomic status is associated with decreasing mammographic density, which is driven by the negative association between BMI and socioeconomic status [21]. Further studies in population-based cohorts of Malay and Indian women are needed.

The lifestyle factors we analysed explained 44.7% of variation in percent density, 16.2% in dense areas and 45.5% in nondense areas respectively. This is consistent with a study in Singaporean Chinese women reported that lifestyle factors explained 13% of the variation in dense areas and 45% in nondense areas respectively [15], but larger than the 25% of variation in percent density reported in that study. One plausible explanation is that there is a wider variation in percent density in our study as it is a multi-ethnic study compared to a Chinese only study.

Of the lifestyle variables that were reported to be associated with mammographic density, our results were not consistent with published literature for hormone replacement therapy and intake of green tea and soy. Hormone replacement therapy, particularly when estrogen is used in combination with progestin, has been consistently shown to be associated with increased density [22,23,24], but this was not observed in our cohort. This may because there were few hormone therapy users in this study [<8%] and the combination that was used was not taken into consideration in our analyses. Intake of soy or green tea was associated with decreased density among Singaporean Chinese women [25], but this was not found in our cohort, perhaps because our cohort was smaller with a lower reported prevalence of daily green tea [7% compared to 10%] and daily soy intake was not quantitated.

There are several limitations to this study. The MyMammo subsidised mammogram programme is an opportunistic screening programme and may not be representative of the population of Malaysia. Subang Jaya, where the recruitment centre is located, is one of the most populous cities in Malaysia and is part of the largest urban agglomeration in Malaysia, the Klang Valley. Therefore the cohort is likely to be more affluent, well-educated and westernised compared to the general Malaysian population. Another limitation is that the cohort does not represent the ethnic distribution of Malaysia and therefore extrapolation to other Asians must be interpreted with care. Malays form the largest proportion (51%) of the Malaysian population, compared to 30% Chinese and 8% Indian [26], but only make up 10% of the cohort. Indeed, few Malays come forward for opportunistic screening mammograms in Malaysia [27] or organised mammography screening in Singapore [28].

In conclusion, our data show that established modifiers of density such as age, BMI, menopausal status and parity do not fully account for the variations observed in percent mammographic density and nondense area among Chinese, Malay and Indian women living in Malaysia. Larger studies investigating genetic and other possible determinants of mammographic density are therefore required in this population.

## Supporting Information

S1 Table Selected characteristics of all (N = 903) and randomly selected Chinese women (n = 205). (DOCX)

S2 Table Linear regression analysis of percent density with lifestyle factors (N = 542). (DOCX)

S3 Table Linear regression analysis of dense area with lifestyle factors (N = 542). (DOCX)

S4 Table Linear regression analysis of nondense area with lifestyle factors (N = 542). (DOCX)

S1 Fig Correlation and concordance plots to evaluate agreement on a continuous measure obtained by ImageJ and Cumulus for 50 images used for validation. (DOCX)

S2 Fig Bland-Altman plots to assess the level of agreement between the two methods; the novel and automated ImageJ method and the established Cumulus method. (DOCX)

S3 Fig Bland-Altman plots to assess the level of agreement for square-root transformed percent density measurements between ImageJ and Cumulus for randomly selected (from left to right) Chinese, Indian and Malay women. (DOCX)

S4 Fig Bland-Altman plots to assess the level of agreement for square-root transformed dense area measurements between ImageJ and Cumulus for randomly selected (from left to right) Chinese, Indian and Malay women. (DOCX)

S5 Fig Bland-Altman plots to assess the level of agreement for square-root transformed breast area measurements between ImageJ and Cumulus for randomly selected (from left to right) Chinese, Indian and Malay women. (DOCX)

S6 Fig Distribution of percent density by ethnicity. (DOCX)
