# Supplementary material for: Ethnic Differences in Mammographic Densities: An Asian Cross-Sectional Study
Source: PLoS One. 2015 Feb 6;10(2):e0117568. doi: 10.1371/journal.pone.0117568 (PMC4320072; doi:10.1371/journal.pone.0117568)
Supplement: S1 Table — (DOCX) [file pone.0117568.s001.docx]

| ***Table S1*. Selected characteristics of all (N=903) and randomly selected Chinese women (n=205)** | | | | |
| --- | --- | --- | --- | --- |
| **Characteristic** | **All (N=903)** | | **Sample (n=205)** | |
|  | **Mean (SD)** | **%** | **Mean (SD)** | **%** |
| Age (years) | 50.8 (7.3) |  | 50.8 (7.9) |  |
| Height (cm) | 1.57 (5.4) |  | 157.2 (5.9) |  |
| Weight (kg) | 58.8 (9.5) |  | 58.6 (10.2) |  |
| BMI (kg/m^2^) | 23.7 (3.6) |  | 23.7 (4.0) |  |
| Parity |  | 84.1 |  | 83.4 |
| Number of FTP | 2.2 (1.3) |  | 2.1 (1.3) |  |
| Menopausal status (post) |  | 44.6 |  | 41.5 |
| Percent density (%) | 31.0 (13.8) |  | 31.7 (13.7) |  |
| Dense area (cm^2^) | 26.7 (12.8) |  | 27.0 (11.8) |  |
| Nondense area (cm^2^) | 64.5 (30.7) |  | 63.6 (30.2) |  |
| Abbreviations: BMI body mass index, FTP full term pregnancy | | | | |
|  | | | | |
