# Supplementary material for: Ethnic Differences in Mammographic Densities: An Asian Cross-Sectional Study
Source: PLoS One. 2015 Feb 6;10(2):e0117568. doi: 10.1371/journal.pone.0117568 (PMC4320072; doi:10.1371/journal.pone.0117568)
Supplement: S2 Table — (DOCX) [file pone.0117568.s002.docx]

|  | | | | |  |  |  |  |  |  |  |  |  |
| --- | --- | --- | --- | --- | --- | --- | --- | --- | --- | --- | --- | --- | --- |
| ***Table S2.* Linear regression analysis of percent density with lifestyle factors (N=542)** | | | | | | | | | | | | | |
| **Variable** | **Unadjusted** | | | **Age and BMI adjusted** | | | | **Multivariable^a^ adjusted** | | | **Multivariable^a^ and ethnicity adjusted, *R^2^*=0.45** | | |
|  | **Estimate** | **SE** | ***P*** | **Estimate** | | **SE** | ***P*** | **Estimate** | **SE** | ***P*** | **Estimate** | **SE** | ***P*** |
| Age (years) | -0.54 | 0.08 | <0.001 | -0.47 | | 0.06 | <0.001 | -0.14 | 0.09 | 0.117 | -0.19 | 0.09 | 0.039 |
| Ethnicity | | | | | | | | | | | | | |
| Chinese | Reference | | | Reference | | | | Reference | | | Reference | | |
| Malay | -8.19 | 1.42 | <0.001 | -4.83 | | 1.21 | <0.001 | -4.24 | 1.19 | <0.001 | -4.24 | 1.19 | <0.001 |
| Indian | -10.18 | 1.29 | <0.001 | -5.09 | | 1.10 | <0.001 | -4.21 | 1.08 | <0.001 | -4.21 | 1.08 | <0.001 |
| Anthropometric variables | | | | | | | | | | | | | |
| Height (cm)^b^ | 0.19 | 0.10 | 0.062 | 0.41 | | 0.08 | <0.001 | 0.42 | 0.08 | <0.001 | 0.38 | 0.08 | <0.001 |
| Weight (kg)^b^ | -0.58 | 0.04 | <0.001 | -0.64 | | 0.04 | <0.001 | -0.63 | 0.04 | <0.001 | -0.57 | 0.04 | <0.001 |
| BMI (kg/m^2^) | -1.61 | 0.10 | <0.001 | -1.56 | | 0.09 | <0.001 | -1.54 | 0.09 | <0.001 | -1.40 | 0.10 | <0.001 |
| Reproductive variables | | | | | | | | | | | | | |
| Age at menarche (years) | 0.27 | 0.44 | 0.535 | 0.16 | | 0.34 | 0.643 | 0.32 | 0.33 | 0.341 | 0.37 | 0.33 | 0.266 |
| Parity (parous vs. nulliparous) | -5.57 | 1.73 | 0.001 | -4.64 | | 1.35 | 0.001 | -4.72 | 1.32 | <0.001 | -4.31 | 1.30 | 0.001 |
| Number of FTP^c^ | -1.59 | 0.52 | 0.002 | -0.49 | | 0.42 | 0.245 | -0.06 | 0.47 | 0.903 | 0.06 | 0.48 | 0.897 |
| Age at first FTP (years)^d^ | 0.52 | 0.13 | <0.001 | 0.26 | | 0.10 | 0.012 | 0.24 | 0.10 | 0.015 | 0.20 | 0.10 | 0.041 |
| Breastfeeding^e^ | | | | | | | | | | | | | |
| Ever | -0.30 | 1.66 | 0.856 | -0.58 | | 1.31 | 0.656 | -0.94 | 1.29 | 0.469 | 0.62 | 1.31 | 0.636 |
| Multiple times ≥12 month each | -4.53 | 1.90 | 0.018 | -3.21 | | 1.50 | 0.033 | -3.04 | 1.50 | 0.043 | -1.81 | 1.56 | 0.246 |
| Duration (months) | | | | | | | | | | | | | |
| 0 | Reference | | | Reference | | | | Reference | | | Reference | | |
| >0-12 | 0.76 | 1.73 | 0.663 | -0.04 | | 1.36 | 0.977 | -0.59 | 1.35 | 0.660 | -0.05 | 1.19 | 0.967 |
| >12 | -2.22 | 1.90 | 0.243 | -1.66 | | 1.51 | 0.272 | -1.62 | 1.50 | 0.279 | -0.60 | 1.59 | 0.704 |
| Menopausal status (post) | -8.19 | 1.14 | <0.001 | -6.32 | | 1.37 | <0.001 | -6.29 | 1.35 | <0.001 | -5.80 | 1.34 | <0.001 |
| Age at menopause (years)^f^ | 0.19 | 0.18 | 0.275 | 0.11 | | 0.15 | 0.480 | 0.07 | 0.15 | 0.645 | 0.05 | 0.15 | 0.758 |
| Bilateral oophorectomy (yes) | -7.91 | 3.18 | 0.013 | -4.60 | | 2.51 | 0.068 | -2.95 | 2.49 | 0.236 | -3.55 | 2.45 | 0.149 |
| Exogenous hormone use | | | | | | | | | | | | | |
| Oral contraceptives use | | | | | | | | | | | | | |
| Ever | -0.68 | 1.32 | 0.607 | -0.48 | | 1.03 | 0.643 | 0.19 | 1.00 | 0.854 | 0.34 | 1.00 | 0.733 |
| Current^g^ | -2.56 | 5.19 | 0.623 | -2.19 | | 4.17 | 0.600 | -1.78 | 4.14 | 0.667 | -2.39 | 4.07 | 0.557 |
| Duration (months) | | | | | | | | | | | | | |
| 0 | Reference | | | Reference | | | | Reference | | | Reference | | |
| 1-11 | 1.05 | 2.28 | 0.645 | 1.33 | | 1.78 | 0.454 | 1.91 | 1.92 | 0.321 | 1.14 | 1.71 | 0.506 |
| ≥12 | -1.30 | 1.47 | 0.380 | -1.12 | | 1.15 | 0.329 | 0.33 | 1.13 | 0.771 | 0.04 | 1.14 | 0.970 |
| Hormone replacement therapy use^f^ | | | | | | | | | | | | | |
| Ever | -5.12 | 2.23 | 0.022 | -0.52 | | 1.79 | 0.771 | 0.32 | 1.74 | 0.854 | 0.47 | 1.72 | 0.786 |
| Current | -3.50 | 3.22 | 0.279 | -1.54 | | 0.14 | <0.001 | -2.56 | 2.63 | 0.331 | -2.07 | 2.64 | 0.433 |
| Duration (months) | | | | | | | | | | | | | |
| 0 | Reference | | | Reference | | | | Reference | | | Reference | | |
| 1-<6 | -2.34 | 3.71 | 0.529 | 0.68 | | 2.92 | 0.817 | 6.63 | 5.15 | 0.198 | 1.01 | 2.81 | 0.718 |
| 6-<24 | -0.09 | 6.15 | 0.989 | 4.11 | | 4.86 | 0.399 | 3.10 | 3.62 | 0.393 | 5.08 | 4.65 | 0.275 |
| ≥24 | -8.03 | 2.98 | 0.007 | -2.41 | | 2.42 | 0.320 | 1.58 | 2.35 | 0.502 | -0.94 | 2.32 | 0.687 |
| Lifestyle variables | | | | | | | | | | | | | |
| Smoking (ever) | 3.03 | 2.20 | 0.169 | 1.35 | | 1.72 | 0.434 | 0.79 | 1.67 | 0.636 | 0.95 | 1.68 | 0.571 |
| Smoking (current) | 2.44 | 4.18 | 0.559 | -0.85 | | 3.27 | 0.794 | -1.69 | 3.18 | 0.596 | -1.08 | 3.16 | 0.732 |
| Alcohol (>once a month) | 1.60 | 1.51 | 0.292 | 0.82 | | 1.19 | 0.487 | 1.07 | 1.15 | 0.350 | 0.45 | 1.16 | 0.698 |
| Coffee (≥one cup a day) | -0.77 | 1.21 | 0.526 | 0.63 | | 0.95 | 0.505 | 1.33 | 0.92 | 0.151 | 1.36 | 0.91 | 0.138 |
| Green tea (≥one cup a day) | 2.14 | 2.61 | 0.411 | 0.32 | | 2.01 | 0.875 | 0.75 | 1.96 | 0.703 | 0.93 | 1.94 | 0.632 |
| Black tea (≥one cup a day) | -3.50 | 1.48 | 0.018 | -1.33 | | 1.14 | 0.244 | -0.95 | 1.11 | 0.394 | -0.25 | 1.13 | 0.824 |
| Soya products (daily) | -3.92 | 1.96 | 0.046 | -1.37 | | 1.54 | 0.376 | -2.18 | 1.50 | 0.146 | -2.09 | 1.48 | 0.158 |
| Average income | | | | | | | | | | | | | |
| <RM5000 | Reference | | | Reference | | | | Reference | | | Reference | | |
| RM5000-10000 | 0.89 | 1.38 | 0.523 | -0.70 | | 1.11 | 0.530 | -0.55 | 1.08 | 0.612 | -0.42 | 1.06 | 0.691 |
| >RM10000 | 3.58 | 1.51 | 0.018 | 1.03 | | 1.21 | 0.398 | 0.92 | 1.19 | 0.439 | 1.06 | 1.20 | 0.378 |
| Educational level |  | | | | | | | | | | | | |
| Primary | Reference | | | Reference | | | | Reference | | | Reference | | |
| Secondary | 6.51 | 2.39 | 0.007 | -0.90 | | 1.97 | 0.648 | -1.27 | 1.94 | 0.514 | -0.33 | 1.93 | 0.866 |
| Tertiary | 8.38 | 2.40 | 0.001 | -0.04 | | 2.05 | 0.985 | -1.41 | 2.08 | 0.498 | -0.10 | 2.10 | 0.963 |
| Other variables | | | | | | | | | | | | | |
| Family history of breast cancer | 0.09 | 1.96 | 0.962 | 1.49 | | 1.53 | 0.332 | 1.25 | 1.49 | 0.400 | 0.82 | 1.48 | 0.578 |
| ^a^ Adjusted for age and variables significantly associated with percent mammographic density (*P* < 0.05) in all multivariable models, i.e., BMI, parity status, mean centred age at first full term pregnancy and menopausal status.  ^b^ Height and weight coefficients are estimated after excluding BMI.  ^c^ The unadjusted and age+BMI adjusted analyses are using actual number of full term pregnancies. For the multivariable models, 1 was subtracted from the number of full term pregnancies (FTP) for parous women and nulliparous women are coded as 0. Refer to Statistical Analysis for details.  ^d^ The unadjusted and age+BMI adjusted analyses are restricted to parous women. For the multivariable models, the mean centred age at first FTP is used. Nulliparous women are coded as 0. Refer to Statistical Analysis for details.  ^e^ Analysis is restricted to parous women.  ^f^ Analysis is restricted to postmenopausal women.  ^g^ Analysis is restricted to premenopausal women. | | | | | | | | | | | | | |
