# Supplementary material for: Ethnic Differences in Mammographic Densities: An Asian Cross-Sectional Study
Source: PLoS One. 2015 Feb 6;10(2):e0117568. doi: 10.1371/journal.pone.0117568 (PMC4320072; doi:10.1371/journal.pone.0117568)
Supplement: S1 Fig — (DOCX) [file pone.0117568.s005.docx]

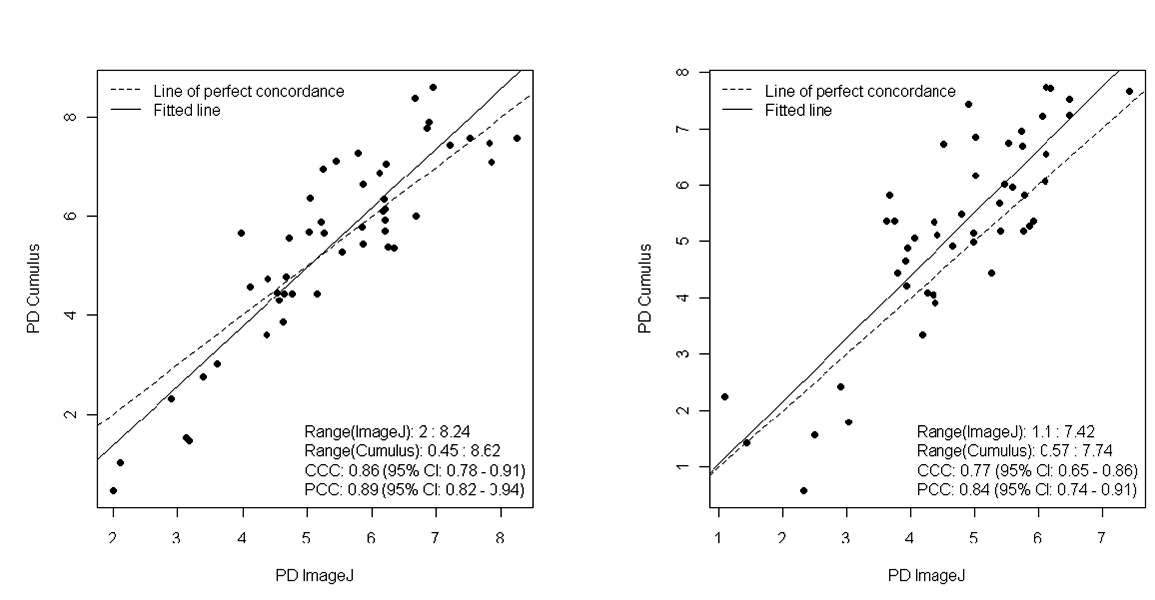
**Supplementary Figure S1:** Correlation and concordance plots to evaluate agreement on a continuous measure obtained by ImageJ and Cumulus for 50 images used for validation. Left: CC; Right: MLO; PD: Percent density; PCC: Pearson’s correlation coefficient; CCC: Concordance correlation coefficient
