# Supplementary material for: Ethnic Differences in Mammographic Densities: An Asian Cross-Sectional Study
Source: PLoS One. 2015 Feb 6;10(2):e0117568. doi: 10.1371/journal.pone.0117568 (PMC4320072; doi:10.1371/journal.pone.0117568)
Supplement: S2 Fig — (DOCX) [file pone.0117568.s006.docx]

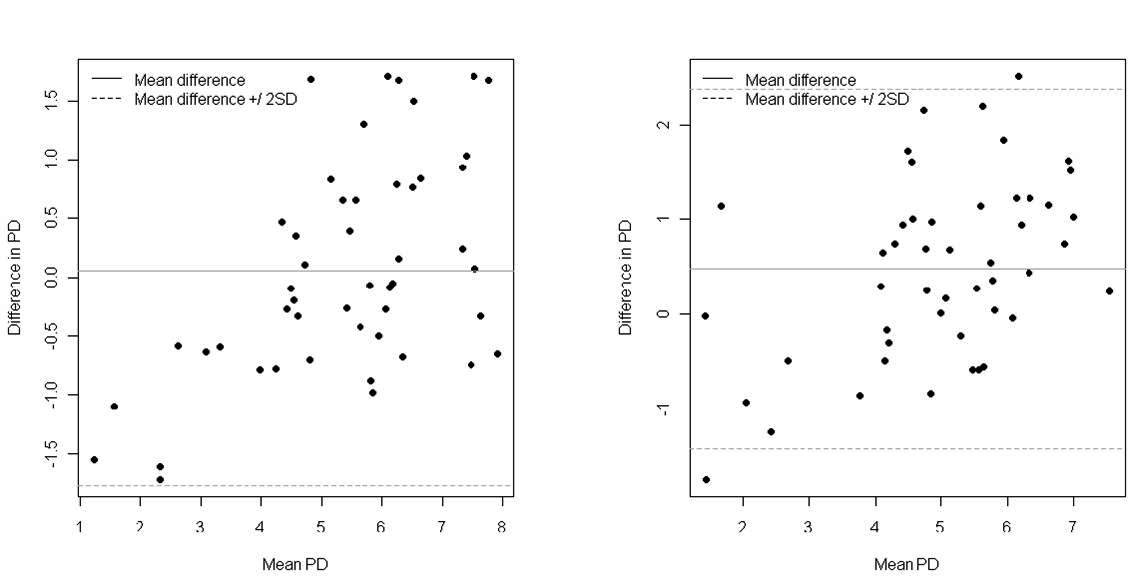


**Supplementary Figure S2:** Bland-Altman plots to assess the level of agreement between the two methods; the novel and automated ImageJ method and the established Cumulus method. Left: CC; Right: MLO. Of the 50 images used for validation of the trained estimates, two fell outside of the mean bias ±2 SD range (dashed lines)*.* The mean difference (solid line) in square-root transformed percent density (PD) between the two methods was less than 0.5. The means of the two measurements were not significantly different (P = 0.133; Welch Two Sample t-test).
