# Supplementary material for: Ethnic Differences in Mammographic Densities: An Asian Cross-Sectional Study
Source: PLoS One. 2015 Feb 6;10(2):e0117568. doi: 10.1371/journal.pone.0117568 (PMC4320072; doi:10.1371/journal.pone.0117568)
Supplement: S3 Fig — (DOCX) [file pone.0117568.s007.docx]

**
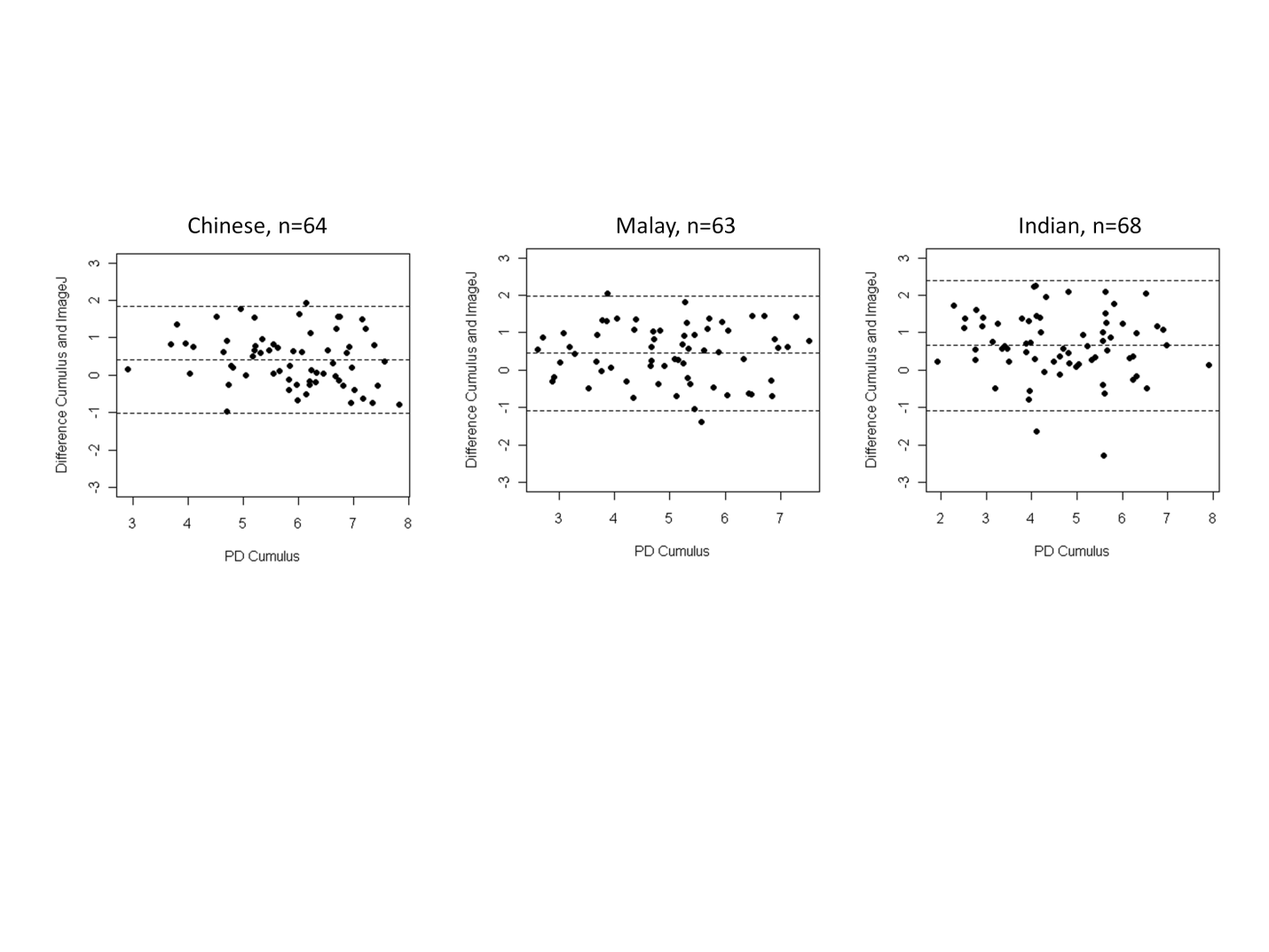
**

**Supplementary Figure S3:** Bland-Altman plots to assess the level of agreement for square-root transformed percent density measurements between ImageJ and Cumulus for randomly selected (from left to right) Chinese, Indian and Malay women. Pearson’s correlation coefficient: Chinese, 0.78; Indian, 0.80; Malay, 0.81.
