# Supplementary material for: Ethnic Differences in Mammographic Densities: An Asian Cross-Sectional Study
Source: PLoS One. 2015 Feb 6;10(2):e0117568. doi: 10.1371/journal.pone.0117568 (PMC4320072; doi:10.1371/journal.pone.0117568)
Supplement: S4 Fig — (DOCX) [file pone.0117568.s008.docx]

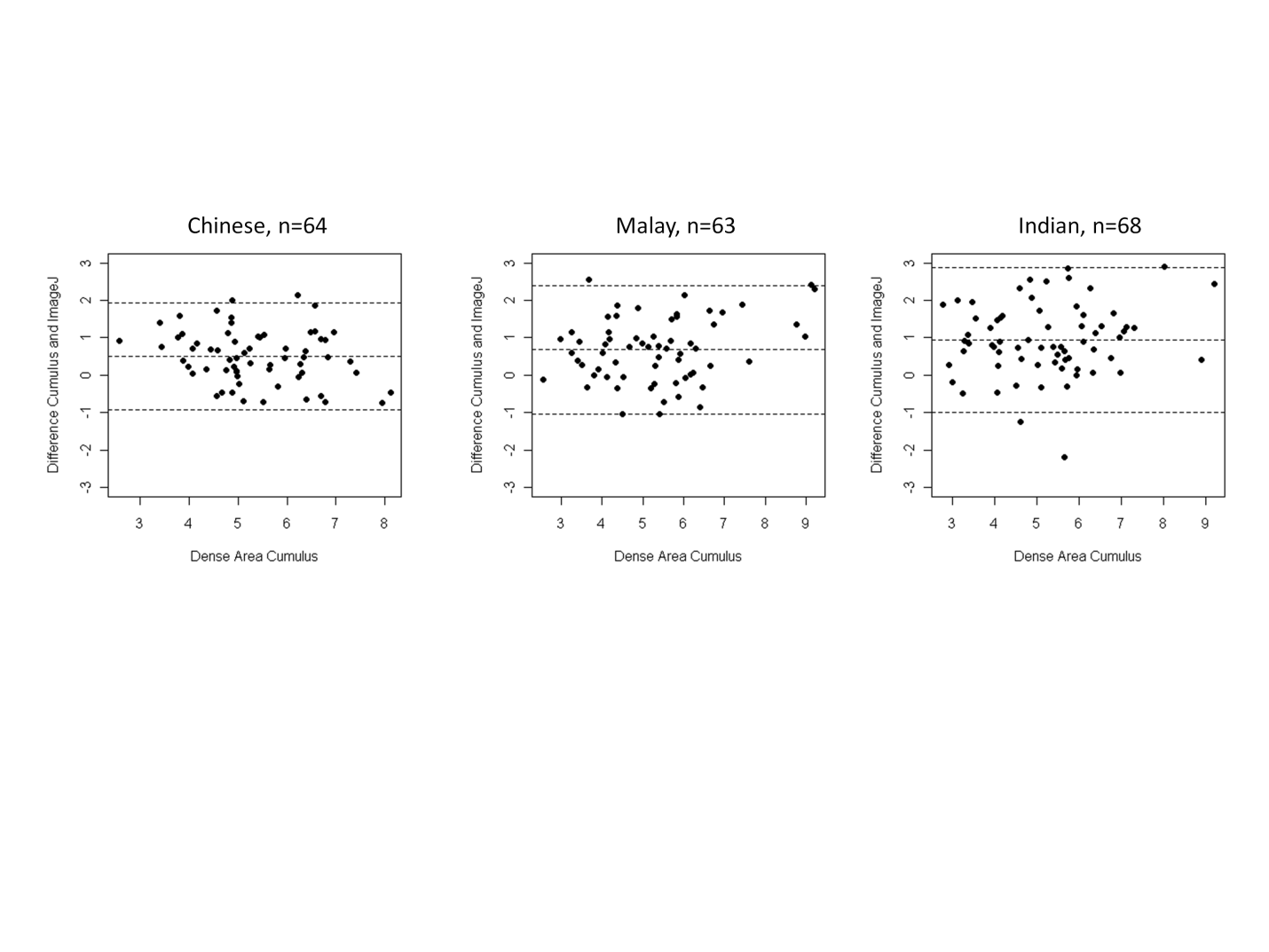


**Supplementary Figure S4:** Bland-Altman plots to assess the level of agreement for square-root transformed dense area measurements between ImageJ and Cumulus for randomly selected (from left to right) Chinese, Indian and Malay women. Pearson’s correlation coefficient: Chinese, 0.82; Indian, 0.80; Malay, 0.87.
