# Supplementary figures and images for: Ethnic Differences in Mammographic Densities: An Asian Cross-Sectional Study
Source: PLoS One. 2015 Feb 6;10(2):e0117568. doi: 10.1371/journal.pone.0117568 (PMC4320072; doi:10.1371/journal.pone.0117568)

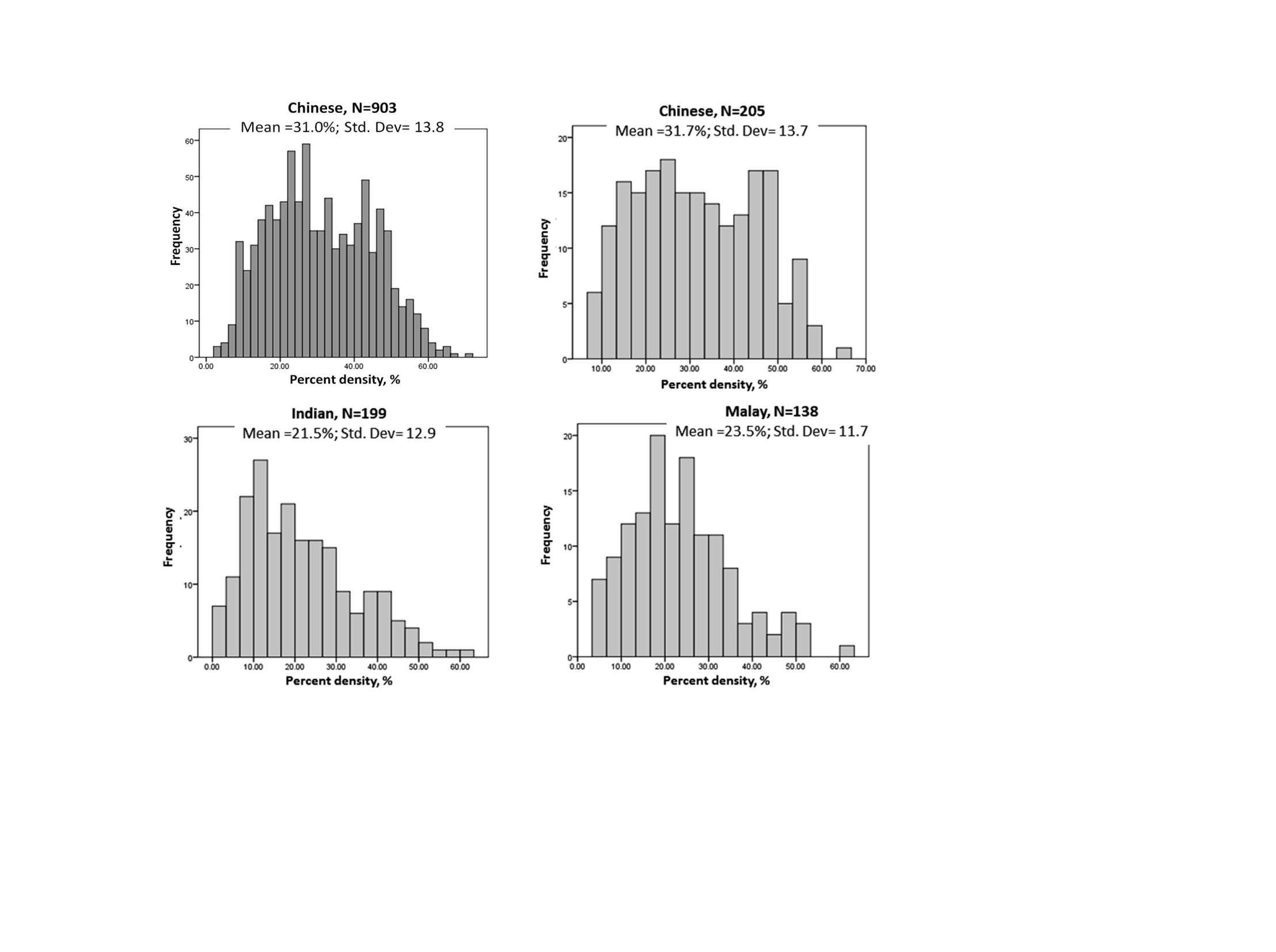


**Supplementary Figure S6:** Distribution of percent density by ethnicity

Supplement: S6 Fig — (DOCX) [file pone.0117568.s010.docx]
